# Childhood Malignant Brain Tumors: Balancing the Bench and Bedside

**DOI:** 10.3390/cancers13236099

**Published:** 2021-12-03

**Authors:** Colin Thorbinson, John-Paul Kilday

**Affiliations:** 1 Children’s Brain Tumour Research Centre, Department of Paediatric Oncology, Royal Manchester Children’s Hospital, Manchester University NHS Foundation Trust, Manchester M13 9WL, UK; Colin.Thorbinson@mft.nhs.uk; 2 The Centre for Paediatric, Teenage and Young Adult Cancer, Institute of Cancer Sciences, The University of Manchester, Manchester M20 4BX, UK

**Keywords:** pediatric, brain, tumor, medulloblastoma, glioma, ependymoma

## Abstract

**Simple Summary:**

Brain tumors remain the most common childhood solid tumors, accounting for approximately 25% of all pediatric cancers. They also represent the most common cause of cancer-related illness and death in this age group. Recent years have witnessed an evolution in our understanding of the biological underpinnings of many childhood brain tumors, potentially improving survival through both improved risk group allocation for patients to provide appropriate treatment intensity, and novel therapeutic breakthroughs. This review aims to summarize the molecular landscape, current trial-based standards of care, novel treatments being explored and future challenges for the three most common childhood malignant brain tumors—medulloblastomas, high-grade gliomas and ependymomas.

**Abstract:**

Brain tumors are the leading cause of childhood cancer deaths in developed countries. They also represent the most common solid tumor in this age group, accounting for approximately one-quarter of all pediatric cancers. Developments in neuro-imaging, neurosurgical techniques, adjuvant therapy and supportive care have improved survival rates for certain tumors, allowing a future focus on optimizing cure, whilst minimizing long-term adverse effects. Recent times have witnessed a rapid evolution in the molecular characterization of several of the common pediatric brain tumors, allowing unique clinical and biological patient subgroups to be identified. However, a resulting paradigm shift in both translational therapy and subsequent survival for many of these tumors remains elusive, while recurrence remains a great clinical challenge. This review will provide an insight into the key molecular developments and global co-operative trial results for the most common malignant pediatric brain tumors (medulloblastoma, high-grade gliomas and ependymoma), highlighting potential future directions for management, including novel therapeutic options, and critical challenges that remain unsolved.

## 1. Introduction

Brain tumors are the most common solid tumors of childhood, accounting for approximately 25% of all pediatric malignancies, and represent the leading cause of cancer-induced morbidity and mortality in this age group [1]. With an incidence of approximately 6 per 100,000 children in industrialized society [2], these tumors represent a spectrum of clinically, pathologically and biologically diverse subtypes which can pose significant challenges in conducting research and clinical trials, necessitating international collaboration.

Over recent decades, cure rates for selected pediatric brain tumors (most notably medulloblastoma) have improved [3], predominantly as a consequence of advances in multiparametric neuro-imaging, neurosurgical techniques, radiation therapy and multiagent chemotherapy, together with improved supportive care. However, such survival advances are typically offset by a therapy-induced toxicity burden for the patient, with wide-reaching consequences for the child, their family and society. Moreover, for the majority of brain tumors, prognosis has remained static for over 30 years despite these technological improvements.

To overcome this impasse, the pediatric neuro-oncology community has shifted focus to develop risk-stratified treatment protocols that aim to reduce iatrogenic morbidity while maintaining outcomes for favorable-risk lesions, and improve cure rates for tumors refractory to conventional therapy, either through intensification or novel agents. This strategy has been supplemented by an evolution in our understanding of the molecular pathogenesis of almost all pediatric brain tumors.

Such molecular advances have identified potential cells of origin, and led to the identification of multiple biologically distinct subgroups within most brain tumor entities, therein allowing accurate risk stratification for affected children when incorporated with clinical, histological and survival data. In addition, oncogenic biological pathways amenable to manipulation using novel targeted agents have been identified. 

This article will provide a summary of the most common malignant pediatric brain tumors (medulloblastoma, high-grade gliomas and ependymoma) with particular focus on inherent molecular advancements and potential future directions for management, including novel therapeutic options.

## 2. Medulloblastoma

### 2.1. Background

Medulloblastoma (MB) represents the most common malignant brain tumor in children, accounting for approximately 20% of all central nervous system (CNS) tumors [2,4]. It also comprises over 60% of intracranial embryonal tumors, a recently characterized entity consisting of atypical teratoid rhabdoid tumors (ATRTs), embryonal tumors with multilayer rosettes (ETMRs), CNS neuroblastoma with *FOX2* alteration and malignant neuroepithelial tumors with *BCOR* alteration [5]. 

Arising within the cerebellum, MBs are observed across all age categories but are most frequently identified at a median age of five years [6]. Demographic, histological and prognostic heterogeneity embody MB, while it represents the first brain tumor where revolutionary global initiatives (such as the Medulloblastoma Advanced Genomics International Consortium (MAGIC)) have transformed our understanding of the molecular underpinnings of MB pathogenesis, enabling improved patient risk stratification to potentially influence clinical outcome [7].

### 2.2. Histopathology

MBs share a primitive embryonal phenotype comprising malignant cells of stereotypic histological patterns, dominated by neuronal antigen expression [8]. World Health Organization (WHO) pathological classification systems have historically divided MB into a classic subtype accounting for 72% of all cases, a desmoplastic/nodular variant of which medulloblastoma with extensive nodularity (MBEN) is a subgroup and a large cell/anaplastic variant which has historically been assigned an adverse prognostic association [5,9].

### 2.3. Molecular Classification

**Figure 1:**
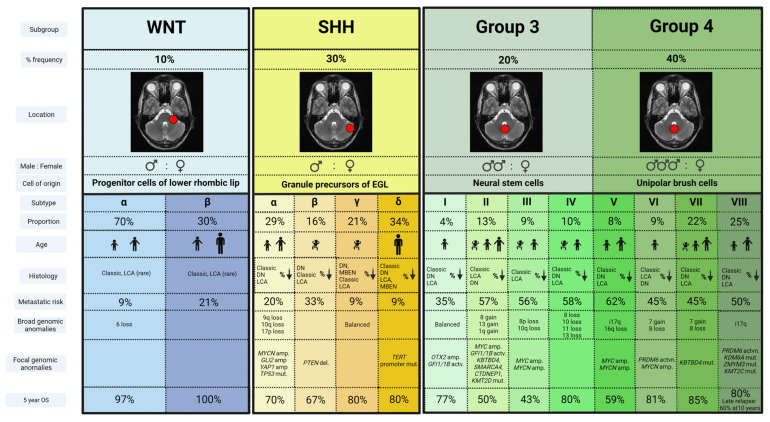
Molecular subgroups and in-group subtypes of medulloblastoma; the four globally recognized molecular subgroups of medulloblastoma (WNT, SHH, Group 3 and Group 4) are shown, together with the current subtypes within WNT and SHH subgroups, as per [13], and Groups 3 and 4, in accordance with [14,15]. Two WNT-activated subtypes are reported, alongside 4 SHH subtypes. Groups 3 and 4 are likely now best considered as a spectrum of 8 different subtypes, each with biological and clinical characteristics. Age-related cartoons depict infant, young child (2–5 years), child (5–12 years), adolescent and older (12+ years). Key: OS = overall survival, DN = desmoplastic/nodular histology, LCA = large cell anaplastic histology, MBEN = medulloblastoma with extensive nodularity, amp. = amplification, mut. = mutation, del. = deletion, and actvn. = activation.

In the past decade, seminal transcriptomic MB analyses led to a global consensus establishing the identification of four discrete molecular subgroups, likely arising from distinct cells of origin—wingless-activated (WNT), sonic hedgehog (SHH), Group 3 and Group 4 MB [10,11]. Further molecular scrutiny of these four groups has now identified somatic mutations targeting chromatin modification as the leading driver for MB heterogeneity via epigenetic dysregulation [12]; further subdivisions have now been established [13,14,15,16] (Figure 1).

#### 2.3.1. WNT Activated (WNT)

WNT MBs account for approximately 10% of all MBs, and often arise in older children with equal gender distribution [11]. Typically occurring in the midline, they frequently invade the lateral recess of the brainstem through the foramen of Luschka, due to a lower rhombic lip cell of origin [17,18]. They rarely metastasize and morphology is typically of the classic variant [8].

Somatic activating mutations in exon 3 of *CTTNB1*, which encodes B-catenin, are found in 80–90% of WNT MB, with 85–90% displaying monosomy 6 [19,20,21,22,23]. Mutations in the adenomatous polyposis coli (*APC*) gene are common in WNT tumors lacking *CTTNB1* mutations [15,24]. Less frequently occurring mutations include *TP53*, *SMARCA4*, *KMT2D* and *DDX3X* [11,15,25,26]. *TP53* mutation occurs only in a minority of WNT MB, and is not prognostic, unlike the SHH subtype [27].

#### 2.3.2. Sonic Hedgehog-Activated-Activated (SHH)

SHH MB represents approximately 30% of all cases, presenting predominantly in a bimodal age distribution; below three years and in young adults [5,8,10]. Originating from granule progenitor cells SHH MBs localize almost exclusively within cerebellar hemispheres [17,28]. All nodular desmoplastic MBs belong to the SHH subgroup, although other histologies can be observed [21,29]. They are most commonly localized at diagnosis and morphology frequently correlates with underlying genetic abnormalities.

SHH MBs are characterized by activation of the SHH pathway as a result of somatic or germline mutations in a number of genes including *SMO*, *PTCH1* and *SUFU* [30]. While *PTCH1* mutations are seen across 30–50% of SHH MBs, *SUFU* and *SMO* mutations are typically seen in infant and adult SHH MBs, respectively [30]. *TP53* mutations typically arise in childhood SHH MBs [27]. Recent epigenomic profiling has identified a further four clinically distinct granular molecular subclasses of SHH MB, alpha, beta, gamma and delta [13]. SHH-alpha MBs predominate in children, whereas infants are most commonly associated with SHH-beta and SHH-gamma, and SHH-delta is typically observed in adult patients [8].

#### 2.3.3. Group 3

Group 3 tumors account for 25% of all MB cases, predominate in males and occur most frequently in younger children between the ages of 2 and 5 years [8]. Thought to arise from neural stem cell origin [31], Group 3 MBs have a short symptom interval and are frequently metastatic at diagnosis with small primary tumors [11,28,32]. 

As with Group 4 MB, Group 3 tumors are not characterized by a signature oncogenic pathway. Nevertheless, Group 3 MBs can be associated with activation of GABAergic and photoreceptor pathways [33,34]. Broad genomic aberrations are a feature, while recurrent somatic nucleotide variants are infrequent [7,12,26]. *MYC* amplification is the most common finding (in approximately 17% of cases) commonly occurring within a complex chromosomal rearrangement at the 8q24 locus, resulting in *MYC*–*PVT1* fusion [7,12,13,34]. The presence of isochromosome 17q, activation of growth factor proto-oncogenes *GFI1* and *GFI1B*, and amplification of transcription factor *OTX2* are also observed [13,15,35].

#### 2.3.4. Group 4

Group 4 tumors represent 35% of all MBs, have a male predisposition and are the dominant molecular subgroup in children of 3 to 16 years of age [8,36]. Similarly to Group 3 MB, they arise in the fourth ventricle and are frequently metastatic at diagnosis, but have a longer symptom interval [11,32].

Genetic abnormalities seen in Group 4 tumors include inactivating mutations of the histone demethylase *KDMS6A* and histone modulator *PRDM6*, tandem duplications of *SNCAIP* and amplifications of *CDK6* and *MYCN* [7,12,25,26,33]. Chromosomal copy number variations include deletion of chromosome 8, 11 or 18p, gain of chromosome 1 or 17q and isochromosome 17q, the most common cytogenetic abnormality in the subgroup [37].

### 2.4. Prognostic Factors

Typical risk-stratification systems for MB incorporate age, extent of tumor resection, and metastatic status to define standard and high-risk cohorts, in turn determining therapy administered. Standard-risk patients are older than 3 years, have undergone gross or near total excision (below 1.5 cm^2^ of residual tumor) with localized disease while remaining patients are classified as high risk. However, these and historical prognostic markers (such as anaplastic morphology) may indeed be surrogates for the underlying MB molecular subgroup, suggesting future stratifications require further refinement.

Pediatric patients with standard-risk WNT-activated MB have an excellent prognosis with a 5 year progression-free survival above 90% following standard therapy. SHH MB demonstrates a range of outcomes. Infant SHH MBs beta and gamma have disparate outcomes, with beta conferring a poor prognosis, and gamma good outcomes [38,39]. *TP53* germline positive SHH MBs confer a poor prognosis with a post-therapy 5 year survival of just 30–40%, particularly when associated with *MYCN* and *GLI2* amplification [40], whereas wildtype SHH MB are associated with a favorable outcome with a 5 year survival of approximately 80% [8,27,30].

Group 3 and 4 MBs also demonstrate variable outcomes, influenced by inherent molecular heterogeneity spanning both groups [14]. For example, Group 3 MB generally carry a poor prognosis, particularly *MYC* amplified cases which are often refractory to conventional therapy [41,42,43], while Group 4 MBs demonstrate a variable prognosis, incorporating favorable-risk MBs harboring chromosome 11 loss or chromosome 17 gain [14]. Infantile Group 4 MBs are infrequent but carry a poor prognosis [44].

### 2.5. Current Management/Clinical Trials

The sequential trial-based addition of adjuvant craniospinal radiotherapy and combination chemotherapy to maximal safe tumor resection has improved survival rates for standard-risk patients immeasurably over the last 50 years and is now the accepted standard of care (Table 1). However, such improved cure rates are achieved at a significant burden to the survivor, with most experiencing chronic neurocognitive and neuroendocrine morbidities [45,46]. While standard-risk patients have benefited from a trial-validated reduction in craniospinal radiotherapy intensity [47] (Table 1), high-risk patients continue to require high-dose radiotherapy (36 Gy) and intensified chemotherapy regimens to maintain a 5 year progression-free survival (PFS) of up to 70% [48,49] (Table 1).

Current trial designs utilize refined patient risk stratifications which incorporate the additional knowledge of molecular MB subgroups. Open standard-risk studies including the Children’s Oncology Group (COG) ACNS1422 (NCT02724579), the North American SJMB12 (NCT01878617) and the European SIOP PNET5 trial (NCT02066220) are assessing whether treatment intensity can be reduced without compromising survival rates for favorable-risk MBs (particularly WNT-activated MBs). 

Caution regarding de-escalation of therapy for WNT-activated MBs is evident from the premature termination of trial NCT02212574 which abandoned craniospinal irradiation for these patients, and a recent retrospective analysis of 93 WNT-activated MBs where relapse was associated with a reduction in the cumulative dosing of maintenance chemotherapy [61].

The PNET5 trial is also assessing the radio-sensitizing effect of carboplatin for non-WNT MB, while SJMB12 is the addition of targeted drug therapy in conjunction with conventional agents for specific molecular subgroups (SHH and high-risk Group 3 and 4 MBs). A European biomarker-driven phase III trial for newly stratified high-risk MB opened to recruitment in 2021. Of interest, post-operative residual tumor is not considered a high-risk feature in this study. The trial incorporates a double-randomized design, comparing the efficacy of hyper-fractionated radiotherapy and additional high-dose chemotherapy against standard radiotherapy, followed by a comparison of multimodal continuation chemotherapy versus single agent temozolomide (EudraCT Number: 2018-004250-17).

Infant MB represents a distinct, intensive chemotherapy-only treatment group [29]. Outcomes for infants with nodular desmoplastic SHH MB can be excellent, although it appears that this requires the inclusion of intrathecal methotrexate in addition to systemic therapy [38,39,62,63]. The COG ACNS0334 study of non-nodular desmoplastic MBs, incorporating both induction and high-dose tandem consolidation cycles of chemotherapy reported 100% survival for metastatic SHH MBs and a survival advantage for the incorporation of methotrexate at induction in Group 3MBs [64].

### 2.6. Novel Therapies

Advances in molecular understanding of MB pathogenesis have also provided the opportunity for the application of subgroup-specific novel targeted therapeutics, notably for SHH MBs. Vismodegib and sonidegib are *SMO* inhibitors that have shown objective responses in pediatric recurrent SHH MB [65,66,67,68,69,70,71]. For most patients, such responses were not sustained, as a result of mutations downstream from *SMO* re-activating the pathway [30]. Another important consideration of this therapy is the association with premature growth plate fusions which has led to modification of the current SJMB12 study [70,71,72]. Agents such as silmitasertib, targeting *SMO* downstream mutations in the SHH pathway, are under evaluation in relapsed SHH MB (NCT03904862). *GLI* inhibition by arsenic trioxide is another area of drug development in SHH MB and early phase pediatric tumor trial data are awaited (NCT00024258). 

For non-SHH tumors, the aforementioned SJMB12 study is evaluating the addition of pemetrexed and gemcitabine to conventional chemotherapeutic agents for high-risk Group 3 and 4 MBs (large cell anaplastic histology, metastatic disease or *MYC*/*MYCN* upregulation) after promising high throughput in vitro drug assay analysis [73]. The CDK4/6-cyclin D-Rb pathway was identified as a potential therapeutic target in xenograft models for non-WNT MB [74]. Other proposed approaches include *HDAC* inhibitors, *PI3K* inhibition and BET-bromodomain inhibition to downregulate *MYC* expression in Group 3 MBs, and *LSD1* inhibition of *GFI1*/*GFI1B* overexpression when present in Group 3 and 4 MBs [75,76,77,78].

Finally, despite the challenge posed by the lack of immunogenic targets in CNS tumors, immunotherapy has been proposed as a potential treatment option in relapsed/refractory MB [79]. Anti-EPHA2, HER2 and IL-13Rα2 chimeric antigen receptor T-cell (CAR-T) therapy has been shown to successfully treat murine Group 3 MBs [80] and early phase trials in children have commenced (NCT03500991, NCT04661384). 

## 3. High-Grade Gliomas

### 3.1. Background

This group encapsulates all malignant lesions of glial origin. Alongside embryonal tumors, pediatric high-grade gliomas (pHGGs) are one of the most common malignant tumor groups of the childhood central nervous system, with a collective incidence of 1.1 per 100,000 children [2]. Despite a paradigm shift in our understanding of pHGG molecular subgrouping being distinct from adult counterparts, and some therapeutic successes for particular entities (such as infant HGG), little progress has been made over recent decades to improve the dismal prognosis; pHGGs account for over 40% of all childhood brain tumor deaths [81]. As a result, they remain the focus of several experimental therapeutic research teams.

### 3.2. Histopathology

The vast majority of pHGGs can be classified as anaplastic astrocytomas (WHO Grade III), or glioblastoma (Grade IV). Historically, a minority of diffuse intrinsic pontine gliomas (DIPGs) were morphologically consistent with diffuse astrocytoma (Grade II), likely resulting from sampling bias. However, the identification of pathognomonic oncogenic mutations in DIPG (particularly in histones 3.1 and 3.3), together with established malignant clinical characteristics, resulted in an amendment to current WHO nomenclature, with DIPGs now classified as diffuse midline gliomas with *H3K27* mutation (Grade IV) [5].

### 3.3. Molecular Classification

**Figure 2:**
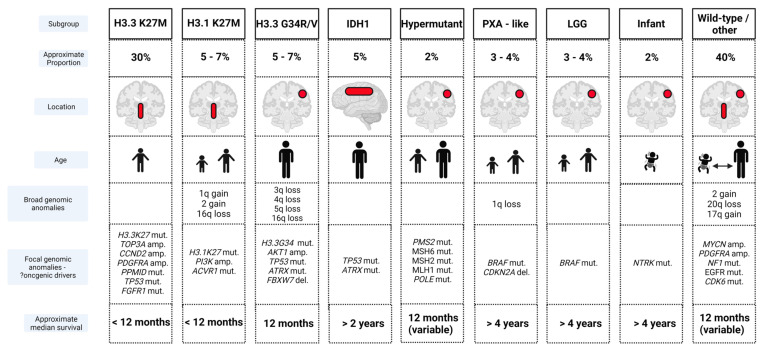
Molecular subgroups of pediatric high-grade glioma. At least nine subgroups are thought to exist, with biological and clinical features highlighted in accordance with [84,90,91]. Age-related cartoons depict infant, young child (2–5 years), child (5–12 years), and adolescent/adult (12+ years). Key: amp. = amplification, mut. = mutation, del. = deletion, IDH = isocitrate dehydrogenase, and PXA, pleomorphic xanthoastrocytoma.

Clear biological distinctions between pHGGs and adult counterparts are now established [82,83], providing a rationale for the failure of many novel therapies derived from adult tumor research. Molecular heterogeneity within pHGGs is also well described [84,85,86,87,88,89,90,91]. The largest molecular meta-analysis of pHGGs published to date, incorporating genomic, epigenomic and transcriptomic profiling has now identified at least nine pHGG subgroups with inherent biological and/or clinical characteristics such as age, tumor location and prognosis [90]. These subgroups express recurrent signature aberrations, which may lead to further refinement of subdivisions in the future (Figure 2).

The predominant pHGG subgroups express mutations of histones *HIST1H3B* (*H3.1*) at position K27, *H3.2* (rarely) and *H3F3A* (*H3.3*) at positions K27 and G34 [90,92]. H3K27M pHGGs are characterized biologically by aberrant expression resulting from loss of trimethylation at lysine 27 on Histone 3 [93,94], and clinically by their midline location (pons, midbrain, thalamus, spina cord) and younger patient age [90,91]. H3.3 G34 subgroup pHGGs are typically located in hemispheric locations, impacting adolescent and older age groups [90,91,95]. The midline location may contribute to the significantly poorer prognosis reported in K27 pHGGs versus G34 counterparts [85,90,91,95], although the mutations alone have been reported as independent prognostic markers in multivariate analysis [90]. Secondary aberrations within the pHGG histone subgroups have also been identified. *TOP3A*, *CCND2*, *PDGFRA*, *PPM1D*, *TP53* and *FGFR1* mutations are more frequently identified in H3.3K27 pHGGs, while H3.1K27 tumors often demonstrate *PI3K* and *ACVR1* mutations and H3.3 G34 pHGGs typically contain *TP53* and *ATRX* mutations [90].

Other subgroups include the IDH mutant pHGGs, associated with a frontal location, an adolescent age range and improved prognosis, hypermutant pHGGs as seen in DNA replication repair deficiency disorders, infant HGGs characterized by *NTRK* mutations and pleomorphic xanthoastrocytoma-like pHGGs and *BRAF* mutated pHGGs, which may represent low-grade lesions that have undergone malignant transformation [90,91,95]. The latter two subgroups may be amenable to novel targeted inhibitor agents and often demonstrate good responses to therapy and improved survival outcomes. A final ‘wild-type’ subgroup comprises pHGGs harboring mutations in genes such as *NF1*, *MYCN*, *EGFR*, and *CDK6* [90].

### 3.4. Prognostic Factors

Prior to the advent of molecular subclassification as described above, the two leading clinical prognostic factors were the extent of surgical resection and tumor histological grade with incomplete resection and Grade IV HGGs conferring a dismal prognosis [96,97]; this continues to be the case today but is supplemented by molecular stratification also. Some studies have also reported a prognostic influence of methylguanine-DNA-methyltransferase (MGMT) expression in the efficacy of temozolomide therapy and patient outcome [54,98].

### 3.5. Current Management/Clinical Trials

The global standard of care for pHGG, the Stupp regimen, stems from adult glioblastoma trial work, which demonstrated that the addition of the alkylating agent temozolomide alongside and after focal radiotherapy, improved progression-free and overall patient survival [99]. Given the molecular disparity between adult HGG and their childhood counterparts, it is therefore unsurprising that temozolomide in a Children’s Oncology Group (COG) pHGG trial analysis (ACNS0126) did not improve outcome compared with previous trials using varied adjuvant chemotherapies [54] (Table 1). However, it remains the standard of care because of the relatively low toxicity profile in comparison to alternative regimens.

The COG ACNS0423 trial noted a marginal outcome benefit for the addition of lomustine with temozolomide [55]; however, it was unclear if this was specific to certain molecular subgroups, while the myelosuppressive toxicity of the regime often proved restrictive. The German Hirntumor (HIT) co-operative group have also reported an improved survival rate for a subset of children with glioblastoma achieving gross total resection compared to historical controls, using an intensified chemotherapy regime alongside and after RT [100].

No definitive therapeutic breakthrough has been made in the treatment of DIPG (now diffuse midline glioma, H3K27 mutant), such that the standard therapy remains radiotherapy alone (Table 1). Modern, multinational collaborative trials, such as the Innovative Therapies for Children with Cancer (ITCC) BIOMEDE study, are developing a more nuanced approach alongside focal RT, utilizing novel inhibitor therapy to target corresponding molecular aberrations present in the lesion (dasatanib, everolimus, and erlotinib) (NCT02233049). Interim overall survival analysis of 193/250 randomized patients concluded that a preferential agent was unlikely to be demonstrated, with survival rates comparable with RT alone, albeit everolimus had the most favorable toxicity profile [58].

### 3.6. Novel Therapies

The paradigm shift in understanding of the molecular heterogeneity of pHGG, together with the failure of conventional therapeutics to significantly improve outcomes for several years, has shifted focus towards developing novel agents that manipulate the epigenetic and genomic aberrations inherent in pHGG molecular subgroups, immunotherapies, and the development of alternative drug administration routes to penetrate the blood–brain barrier such as convection enhanced delivery for diffuse midline glioma H3K27 mutant/DIPG [92,101,102,103,104,105].

Success of mutational target inhibition in specific pHGG subgroups gives credence to this new therapeutic standpoint. For pHGGs with *BRAF V600E* mutations, BRAF inhibitor (BRAFi) activity has been demonstrated as salvage therapy [106,107,108,109]; international co-operative studies are recruiting (NCT03919071). Similar findings of efficacy have been made with neurotrophic tyrosine receptor kinase inhibitor agents for infant HGGs [110,111] and immune checkpoint inhibition in hypermutant pHGGs resulting from replication repair deficiency disorders [112,113,114,115]. Follow-up co-operative early phase trials are now open (NCT04267146, NCT04323046 and NCT04655404).

With respect to the other main subgroups, targeting histone modification is a therapeutic research focus for the H3.1–3.3 pHGG subgroup. Histone deacetylase inhibitors (HDACi) such as panobinostat, vorinostat and valproic acid have been postulated to improve the therapeutic landscape for this subgroup following successful HDACi in vitro pHGG studies, but translational results to date have proved disappointing [116,117,118,119]. Other agents being looked at for this subgroup include ACVR1/ALK inhibitors [120,121] and the imipridones incorporating agents such as ONC201 [122,123,124].

For the IDH mutant pHGG subgroup, blood–brain barrier penetrant IDH inhibitors have been developed for glioma trials (NCT02273739, NCT03343197, NCT02073994 and NCT04056910). These may be specific to IDH-1 (ivosedinib), IDH-2 (enasidenib) or both (vorasidenib). In addition, the use of PARP (poly-adenosine 50-diphosphate-ribose) inhibitors alongside temozolomide as a radiosensitizer is being explored [125].

Immunotherapeutic strategies other than checkpoint blockade are also being evaluated in pHGGs, including cancer peptide vaccine therapy with antigens such as Ephrin A2 (EphA2), interleukin 13 receptor alpha 2 (IL13Ra2), survivin and HLA-A2 (NCT01130077) [126,127,128], autologous dendritic cell vaccine therapy [129], and chimeric antigen receptor (CAR)-T therapy where studies are recruiting (anti-IL13aR2; NCT02208362, anti-GD2; NCT04196413, anti-B7 H3; NCT04185038).

## 4. Ependymoma

### 4.1. Background

Ependymoma is the second most common malignant brain tumor entity in children, after medulloblastoma, representing approximately 10% of all childhood CNS tumors [130]. Most cases present in patients aged below five years and have a male predominance (male: female ratio 0.23: 0.17) [130,131]. Although able to arise anywhere in the neuraxis, over 90% of pediatric ependymomas are intracranial (IC) in origin. Of these, two-thirds occur in the posterior fossa (PF), with the remaining one-third located in the supratentorial (ST) compartment [132]. Leptomeningeal metastasis is uncommon, reported in 2–20% of cases [133,134].

No inherited disorders are consistently reported to predispose to IC pediatric ependymomas. Neurofibromatosis type 2 appears to be associated with the development of spinal ependymomas but typically in the adult population [135].

### 4.2. Histopathology

Current histological classification of ependymoma remains according to the current WHO grading scheme, resulting in four main histological subgroups: subependymoma and myxopapillary ependymoma (grade I), classic (grade II) and anaplastic (grade III) [120]. Subependymoma typically arise in the ventricles of adults, while myxopapillary ependymoma occur exclusively in the spine [5,136]. Consequently, classic and anaplastic variants typically account for all pediatric IC ependymomas. Morphologically they are both characterized by the tumor cell formation into true rosettes (around a canal) or pseudorosettes (around a blood vessel) while anaplasia is signified by increased mitotic figures, necrosis, microvascular proliferation, and an increased an increased cellular nucleus/cytoplasmic ratio [5]. Common immunohistochemical findings include positive staining for glial fibrillary acid protein (GFAP), expression of EMA, S100 and vimentin [5,137].

The utilization of histological grading as a prognostic marker has failed to consistently be of value, in part due to the subjective nature of grade assignation and tumor heterogeneity. These factors, alongside improved understanding of the genomic landscape of pediatric ependymoma, has led to the Consortium to Inform Molecular and Practical Approaches to CNS Tumor Taxonomy (cIMPACT) to recommend that the WHO adopt a new, integrated histological/biological classification system for ependymomas [138].

### 4.3. Molecular Classification

**Figure 3:**
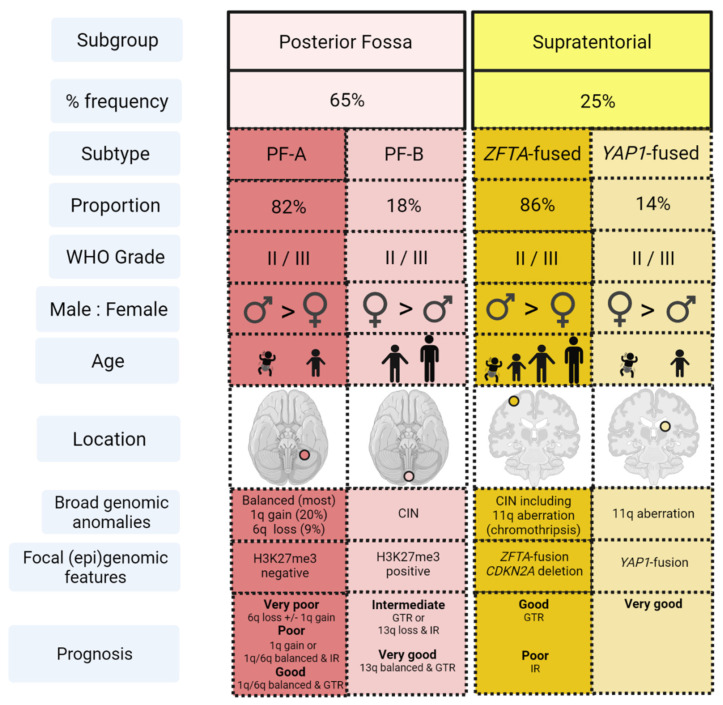
Predominant molecular subtypes of pediatric intracranial ependymoma. Posterior fossa and supratentorial childhood ependymomas are shown, further categorized into four in-group subtypes; PF-A, PF-B, *ZFTA*-fused and *YAP1*-fused. The clinical and biological characteristics of these subtypes are shown, in accordance with [59,120,139,140,141,142,143,144,145,146,147,148,149,150,151,152,153]. Nine molecular subtypes of ependymoma are reported but the remaining subtypes occur in either the spinal cord (spinal subependymoma, spinal myxopapillary ependymoma, spinal ependymoma) or the adult brain (subependymoma: PF and ST) so are not depicted in this figure. Age-related cartoons depict infant, young child (2–5 years), child (5–12 years), adolescent/adult (12+ years). Key: WHO = World Health Organization, CIN = chromosomal instability, GTR = gross total resection, IR = incomplete resection.

Genomic and methylomic profiling of ependymoma has revealed nine distinct molecular subtypes, four of which account for most pediatric IC ependymoma across the PF (PF-A and PF-B) and ST (ST-ZFTA and ST-YAP) compartments [139] (Figure 3).

PF-A ependymomas are biologically characterized by epigenetic dysregulation of DNA methylation and histone modification, often accompanying lack of *H3K27* trimethylation [147,149,150]. With the exception of some genomic imbalances, namely 1q gain and 6q loss, they typically demonstrate a balanced genome [139,147,149]. They are most common in infants and young children, have a tendency towards infiltration, dissemination and consequent poor prognosis [153]. Due to their predominant lateral location and inherent invasiveness, gross total resection (GTR) is often difficult to achieve and therefore relapse rates are high [154]. PF-B ependymomas are characteristically enriched with numerous cytogenetic abnormalities and are more common in adolescents and young adults [139,152,155]. They originate in the midline yet are often amenable to surgical resection, have a low metastatic potential and therefore have a superior outcome to PF-A tumors [139,152,155]. Recent methylation profiling work to further categories these two PF subgroups have reported two major subgroups, nine minor PF-A subtypes and five PF-B subgroups displaying variable clinical and genetic heterogeneity [140,156].

Greater than 70% ST ependymomas contain a zinc finger translocation associated (ZFTA, previously C11Orf95) gene fusion, most commonly RELA-ZFTA and are termed ST-RELA or, more recently ST-ZFTA [139,143,151]. This subtype is found in children and adults, but rarely infants and is often located in frontal or parietal lobes, often with intra-tumoral hemorrhage, cysts or necrosis [157]. ST-YAP is the remaining molecular subgroup, characterized by the fusion of the *YAP1* oncogene with *MAMLD1* [142]. ST-YAP tumors typically arise in ventricular or periventricular locations among infants [142]. Up to 15% of ST ependymoma may not harbor a *RELA* or *YAP1* fusion [158].

### 4.4. Prognostic Factors

Interest remains in identifying prognostic markers to aid patient risk stratification for future ependymoma trial design to improve upon the relative poor long-term outcomes that exist. Akin to medulloblastoma, several clinical and histological putative markers (location, age, tumor grade) have been rendered obsolete by the identification of molecular subgrouping.

The most consistent clinical marker is the extent of surgical resection, with some studies reporting a 60% difference in survival between cases of complete and incomplete tumor resection [59,120,132,159,160,161,162]. The positive prognostic effect of complete excision is maintained across molecular subgroups [120,139].

The infiltrative nature, localization and predisposition to metastasis suggests PF-A ependymomas should exhibit a poorer prognosis when compared with PF-B counterparts, an assumption supported by a retrospective analysis 820 patients with PF ependymoma across four independent cohorts [161]. The recent prospective Children’s Oncology Group (COG) ACN0121 clinical trial, however, found no difference between PF-A and PF-B patient survival, although likely reflecting a paucity of PF-B cases [59]. The study did identify an adverse association with 1q gain in PF-A cases, with survival as low as 30% despite tumor resection and radiotherapy administration [59]. As stated above, tumor gain of chromosome 1q and loss of chromosome 6q are the most commonly observed chromosomal imbalances in ependymoma and appear adverse prognostic factors [59,120,139,141,144,145,146,148,152]. A recent retrospective molecular profiling study of 212 primary PF-B ependymomas identified loss of 13q as a potential novel adverse marker [140].

A retrospective cohort study of 122 ST ependymomas identified *ZFTA*/*RELA* fusion as a poor prognostic marker, regardless of the attainment of resection status, with 10 year PFS and overall survival (OS) of approximately 20% and 50%, respectively [139]. The same study conversely identified excellent ST-YAP1 survival rates of 100% [139]. Nevertheless, data from the ACNS0121 clinical trial failed to show any adverse prognostic implication for ST molecular subgroups, again potentially influenced by the case numbers involved [59].

### 4.5. Current Management/Clinical Trials

The globally accepted standard for pediatric IC ependymomas is maximal, safe surgical resection followed by involved field adjuvant radiotherapy (RT), dosed at 54–59.4 Gy, founded from a 2009 St Jude’s Children’s Research Hospital single-center study of 107 children, demonstrating a 7 year PFS of 77% and OS of 85% [160]. Exceptions to this are in metastatic cases where craniospinal radiotherapy is typically utilized for older children, and infant IC ependymomas, where a chemotherapy only strategy is reserved in order to avoid or delay radiotherapy to the developing brain, with eligibility thresholds of 12 to 18 months for PF tumors and up to 3 years for ST tumors.

Concerns regarding radiotherapy-induced neurotoxicity in young children have resulted in IC ependymoma being the most common pediatric tumor treated with proton beam radiotherapy. By reducing radiation exposure to healthy tissue while delivering therapeutic doses, this modality delivers comparable disease control to modern photon radiotherapy without unexpected toxicity [163,164,165]. Data continue to be collated on latent toxicity [164].

Recent, large international co-operative IC ependymoma trials have been designed to validate the findings of the 2009 St. Jude’s study, evaluate the utility of an aggressive surgical approach, and verify a therapeutic role for chemotherapy either pre or post-radiotherapy (Table 1), since historical data have proven contradictory and inconclusive. The North American CCG-9924 study reported a PFS benefit from immediate post-operative chemotherapy prior to radiotherapy in patients where over 90% of the tumor has been resected [166]; however, this approach has been rebutted by other trial groups [167]. Similarly, outcomes from chemotherapeutic, radiation-sparing strategies for infants have been inconsistent and ultimately disappointing for the majority of children, with only a minority ultimately sparing radiation [168,169,170,171].

The COG ACNS0121 trial confirmed the efficacy of an aggressive surgical approach followed by immediate post-operative radiotherapy, even for children below 3 years of age when compared to historical controls [59]. Long-term follow-up of these younger patients is eagerly awaited. The impact of post-operative chemotherapy to facilitate second-look surgery could not be determined. The COG ACNS0831 study followed on from ACNS0121, with the randomized addition of continuation chemotherapy (vincristine, cisplatin, cyclophosphamide and etoposide) for children treated with adjuvant focal RT following a complete or near total resection [60]. An interim “as treated” analysis of patients was undertaken due to significant non-compliance in patients randomized to receive chemotherapy. This reported a survival advantage for patients receiving chemotherapy (3 year EFS 80% vs. 71%; 1-sided *p*-value = 0.0121) [60].

The open phase II/III SIOP-Europe Ependymoma II trial (NCT02265770) has design similarities with the COG studies, making compliance with post-irradiation chemotherapy randomization imperative to validate the findings from ACNS 0831. Through patient allocation to three strata, the trial also attempts to evaluate the value of pre-radiotherapy chemotherapy and a 8 Gy radiotherapy boost in cases of incomplete resection, and the addition of a of a histone de-acetylase (HDAC) inhibitor, sodium valproate, for infants receiving one year of conventional multiagent chemotherapy.

### 4.6. Novel Therapies

Several biological models and patient derived xenografts have been developed to recapitulate ependymoma subgroups in order to identify new therapeutic targets and test novel therapies [172,173,174]. High throughput drug screening in murine models of *ZFTA* fusion-negative supratentorial ependymoma, characterized by the *Ephb2* oncogene identified 5-fluoracil (5-FU) as a potential active drug against this subtype [174,175]. Fibroblast growth factor receptor inhibitors have also been shown to have activity against patient derived ependymoma cell models and demonstrate efficacy in the clinic [176]. As detailed above, the use of histone deacetylase inhibitors as differentiation therapy is currently under evaluation in the current SIOP-Europe trial, following in vitro analyses [177,178]. Similarly, the phase I/Ib COZMOS trial is evaluating the DNA methyltransferase inhibitor 5′Azacitidine in combination with carboplatin, on the premise that inhibition of aberrant DNA methylation will have therapeutic benefit (NCT03206021). Other novel therapies being explored include chimeric antigen receptor T-Cells (HER2; NCT03500991), based on encouraging pre-clinical murine work [80] and metronomic antiangiogenic therapy [179,180].

## 5. Conclusions

This review exposes the need for the pediatric neuro-oncology community to address the disparity that has developed between advances at the bench compared to the bedside. The potential for an era of biology driven patient care clearly exists yet, at present, international clinical trials struggle to keep pace with the scientific progress made to date. Indeed, many are being rendered outdated before they open to recruitment when evaluated against current molecular advances. This challenge is not unsurmountable and indeed should be embraced as recent years have demonstrated a paradigm shift in our understanding of the molecular pathogenesis across principal malignant brain tumor groups, therein serving as the foundation for developing both risk stratification systems and novel agents as part of the next generation of clinical trials. Nevertheless, results from the review highlight that the statistical design, regulatory infrastructure and ultimately funding of such studies will need urgent consideration to achieve these objectives.

### 5.1. Clinical Trials and Therapeutic Protocols

We have shown that for pediatric medulloblastoma, the four established intrinsic molecular subgroups have now been superseded by the identification of up to 14 subtypes, each demonstrating a disparate corresponding clinical profile. In contrast, most treatment protocols over the past 20 years have continued to treat MBs with the historical backbone of craniospinal radiotherapy and multiagent chemotherapy, only recently tailoring therapy intensity according to WNT/non-WNT subgrouping, without particular focus on the three other subgroups. Encouragingly, open international trials are now attempting to stratify patients and adapt therapy according to molecular diversity. For example, the SIOP-Europe PNET5 study is following a risk-adapted treatment stratification according to low and high-risk WNT subgroups, the SHH-alpha MB subtype (which demonstrate *TP53* mutations), standard-risk biological profiles (including *MYCN* amplified Group 4 MB) and children with a germline mutational profile (NCT02066220). The SJMB12 trial, in addition to evaluating treatment de-escalation for WNT-subgroup patients, is assessing the addition of smoothened inhibitor Vismodegib for SHH MB, and the incorporation of gemcitabine and pemetrexed for high-risk Group 3 and 4 MB patients (NCT01878617). Finally, the SIOP-Europe high-risk medulloblastoma trial is using molecular screening to identify appropriate cases for increased-intensity treatments, including *MYC*/*MYCN* amplification (excluding *MYCN* amplified Group 4 MB) and SHH-alpha MB (EudraCT Number: 2018-004250-17).

Attempts to integrate molecular pathogenesis to inform on therapeutic stratification for most childhood high-grade gliomas or pediatric intracranial ependymoma unfortunately lag significantly behind the progress observed with medulloblastoma. As shown in this review, there is now compelling evidence that molecular subgrouping alone is an independent survival marker for childhood ependymoma, while prognostic adversity is further conferred by the presence of genomic aberrations including chromosome 1q gain and 6q loss in PF-A ependymomas, and potentially 13q loss in PF-B ependymomas. Despite this, international ependymoma clinical trials continue to risk stratify children according to the clinical parameters of patient age and resection status alone; an omission that will require addressing in future clinical trial strategies. With the exception of BIOMEDE 1, large-scale international pediatric HGG trials have also not incorporated biologically derived therapeutic stratification systems, principally because the finding that HGGs encompass an array of discrete subtypes is a relatively recent discovery.

As with medulloblastoma, the observation of up to 14 discrete molecular subtypes of PF ependymoma, at least 3 subtypes of ST ependymoma and up to 10 pediatric HGG subtypes clearly presents a challenge for future trial design. As can be seen from Table 1 of this article, the duration of an international pediatric brain tumor trial can take up to 10 years to complete patient accrual, and even longer to publish data. In order to tailor therapeutic intensity or introduce novel agents against the array of specific tumor subtypes now published in this review, future trials will require novel statistical designs that embrace truly global collaboration to generate timely, rigorous results as increasing molecular subcategorization will lead to significantly smaller patient subpopulations from which statistically sound conclusions must be drawn. Such collaborative efforts may also support less affluent countries to provide equity in diagnostic and therapeutic approaches. Duration of follow-up for specific patient populations will also need to be considered, as evidenced by the high proportion of late relapses in Group 3/4, subtype VIII MB and some non PF-A subgroups of ependymoma.

### 5.2. Conventional and Novel Therapies

While advances in adjuvant therapy have undoubtedly improved the survival of children with malignant brain tumors, the ‘one-therapy-fits-all’ paradigm fails to reflect and tailor to the diverse molecular landscape now apparent. As highlighted by this review, integrating clinical and biological data to generate risk-adapted treatment stratifications can potentially modify conventional therapy intensity and enable the introduction of novel agents.

De-escalation of radiotherapy dosing is being evaluated in several of the current international medulloblastoma clinical trials highlighted in the review. However, such an approach could also be considered for other molecularly-defined tumor entities including Group 4 (often subtype IV) medulloblastomas with chromosome 11 loss, completely resected ST-YAP1 ependymomas, completely resected PF-B ependymomas without 13q loss, and ‘infant’ or ‘LGG-like’ pediatric HGGs. Clearly, any de-escalation of therapy must be approached with extreme caution, as evidenced by the failure of trial NCT02212574 for WNT-activated MB, where a post-operative chemotherapy only strategy led to unacceptable relapse rates.

For some unfavorable-risk tumors, the option of increasing treatment intensity is a possibility as evidenced by current high-risk medulloblastoma trial strategies; however, any trial adopting this approach should consider incorporating disability or health status outcome measures, as they will help determine the quality of potential survivorship afforded [181]. The efficacy of chemotherapy in pediatric ependymoma remains contentious but a potential option to explore for escalation of therapy in certain cases (for instance PF-A tumors with chromosome 1q gain or 6q loss). The interim analysis results of the COG ACNS0831 trial suggested a potential survival advantage for children receiving continuation chemotherapy following tumor excision and post-operative irradiation, yet this requires validation ideally by the open phase II/III SIOP-Europe Ependymoma II trial. The administration of conventional chemotherapy agents and novel agents by alternative means, such as convection enhanced delivery to overcome the blood–brain barrier in diffuse midline glioma, H3K27M pediatric HGGs is also under consideration.

Parallel to modifying the intensity or administration of conventional therapies for childhood malignant brain tumors, much hope rests on establishing novel agents to target aberrant molecular aberrations underpinning tumorigenesis. This review highlights many of the developments in this field across medulloblastoma, pediatric high-grade gliomas and ependymomas. International trial outcomes are awaited for medulloblastoma subgroup-targeted therapy in SJMB12 and combination HDACi therapy across infants in the SIOP Ependymoma II study, while the success of BRAFi and NTRKi in certain pHGG subtypes and the evolving array of targeted primary treatment options for pediatric low-grade glioma give cause for optimism.

While encouraging, challenges nevertheless remain. As described in this review, novel agents against malignant brain tumors are being evaluated in early-phase pediatric studies, yet few successful candidates targeting the spectrum of molecular subtypes that now exist have been identified. One explanation for this is that many early-phase neuro-oncology trials in children assess novel agents in the relapse setting, rather than as primary therapy. In turn, this could potentially generate misleading results on drug efficacy, as evidenced by pre-clinical relapsed medulloblastoma work implicating clonal selection as a potential cause for the disappearance of targetable aberrations between patient-matched primary and relapsed tumors [182,183]. However, the paucity of effective novel agents also reflects the ongoing need for improved pre-clinical models that accurately replicate the specific human disease subtype interrogated, including appropriate immunocompetent murine models to test potential immunotherapies. A further explanation is that many pediatric malignant brain tumors appear driven by epigenetic dysregulation such that tumors rarely harbor immediately actionable mutations, or display significant molecular heterogeneity making resistance to single agent targeted therapy anticipated, as is described for SHH-activated medulloblastoma [30]. Consequently, it is presumed that combination therapy, utilizing novel agents alongside conventional modalities, will better enable local and disseminated disease control rather than a single agent approach in future studies.

### 5.3. Future Challenges

This review highlights the molecular heterogeneity across the most common pediatric malignant brain tumors, together with its relevance to current diagnostic and therapeutic protocols, and strategies to correct the consequent imbalance that arises from bench to bedside. The tumor groups discussed in this review have key clinical challenges that now warrant focus, including intensification or novel combination therapy for unfavorable-risk tumors, de-escalation of intensity for favorable-risk lesions, the treatment of relapse, and a reduction in morbidity, disability and late effects (Table 2). It is now incumbent on the neuro-oncology community to meet and overcome these challenges; in an age of digital technology and social media, where the latest global scientific breakthroughs are acknowledged promptly in the public domain, the families of our patients are demanding this of us.

## Figures and Tables

**Table 1:** Multinational collaborative clinical trials in pediatric medulloblastoma, high-grade gliomas and ependymoma, published since 2000.

Year	Trial	Treatment Strategy	Inclusion Criteria	No. Patients	Results
Medulloblastoma					
1992– 2000	SIOP PNET III [50]	Randomization Arm 1: RT alone (35 Gy CSI + 20 Gy PF boost) Arm 2: 4 cycles alternating Carbo/VP16 and Cyclo/VP16 followed by RT	Age 3–16 yrs Standard-risk MB	179	5 yr EFS 59.8% vs. 74.2% RT + chemotherapy superior
1996– 2000	COG A9961 [3]	Radiotherapy: 23.4 Gy CSI + 32.4 Gy PF boost + weekly VCR Continuation chemotherapy randomization: Arm 1: CCNU/Cis/VCR Arm 2: Cis/Cyclo/VCR	Age 3–21 yrs Standard-risk MB	421	10 yr EFS 74% vs. 78% None superior
2001– 2006	HIT-SIOP PNET-4 [51]	Radiotherapy randomization Arm 1: HFRT (36 Gy CSI, 24 Gy PF boost, 8 Gy TB boost) Arm 2: STRT (23.4 Gy CSI, 30 Gy PF boost) Continuation chemotherapy 8 cycles Cis/CCNU/VCR	Age 4–<22 years Standard-risk MB	340	5 yr EFS 77% vs. 78 None superior
2004– 2016	COG ACNS0331 [52]	Radiotherapy *Children aged 3–7 years randomized:* Randomization 1: CSI: Low-dose (LDCSI) 18 Gy vs. Standard dose (SDCSI) 23.4 Gy Randomization 2: Involved field RT boost vs. Standard volume boost *Children ≥ 8 yrs receive CSI 23.4 Gy, then randomized:* Randomization 3: Involved field RT boost (IFRT) vs. Arm 2: Standard volume boost (PFRT) Continuation chemotherapy 9 cycles (6 × CCNU/Cis/VCR, 3 × Cytoxan/VCR)	Age 3–<21 yrs Standard-risk MB	513	5 yr EFS/OS LDCSI 72.1%/78.1% SDCSI 82.6%/85.9% LDCSI higher event rates and worse survival PFRT 80.8%/85.2% IFRT 82.2%/84.1% None superior
1990– 1996	POG 9031 [49]	Arm 1: 3 cycles Cis/VP16, followed by RT (CSI 35.2–44.0 Gy, PF dose 53.2–54.4 Gy) then 7 cycles VCR/Cyclo continuation chemotherapy Arm 2: RT (CSI 35.2–44.0 Gy, PF dose 53.2–54.4 Gy) followed by 3 cycles Cis/VP16 and 7 cycles VCR/Cyclo continuation chemotherapy	Age 3–18 yrs High-risk MB	224	5 yr EFS/OS: 66%/73.1% vs. 70%/76.1% None superior
1996– 2007	SJMB96 [48]	Radiotherapy Risk Stratified: SR: 23.4 Gy, 36 Gy PF dose and 55.8 Gy TB dose; HR: 36–39.6 Gy and 55.8 Gy TB dose (50.4 Gy dose to metastatic sites) Chemotherapy 4 × Cis/Cyclo/VCR with stem cell rescue	Age 3–20 yrs Standard and High-risk MB	134	5 yr EFS/OS: SR 83%/85% HR 70%/70%
2007– 2017	SJYC07 [38]	Induction chemotherapy LR and IR: MTX/VCR/Cis/Cyclo HR: MTX/VCR/Cis/Cyclo + Vinblastine Consolidation therapy LR: 2 cycles Carbo/Cyclo/VP16 IR ≥ 12 mths old: Focal RT (54 Gy TB dose); IR < 12 months old: 2 × cycles Carbo/Cyclo/VP16 HR < 3 years old: Topo/Cyclo (8 weeks); HR ≥3 years old: could opt for CSI (23.4–39.6 Gy) Continuation chemotherapy All Groups: 6 cycles oral Cyclo/Topo/Erlotinib	Age < 3 yrs newly diagnosed MB OR Age 3–5 yrs -non-metastatic -no high-risk features	81	LR: 1 yr EFS 78.3%, (accrual suspended as EFS below stopping rule). 5 yr EFS/OS: LR 55.3%/85.9% IR: 24.6%/52.8% HR: 16.7%/41%
2013– 2016	ACNS1221 [39]	Induction chemotherapy 3 cycles Cyclo/VCR/MTX/VP16/Carbo Reassessment CR/CCR: No further treatment PRD: Second look surgery + 2 cycles Cyclo/VCR/Carbo/VP16	Age < 4 yrs Localized ND or MBEN	25	2 yr PFS/OS 52%/92% Failed to achieve 2 yr PFS target of 90%; study closed early
2007– 2018	ACNS0332 [53]	Randomization Arm 1: Standard treatment (CSI 36 Gy, PF 55.8 Gy + 6 cycles Cis/Cyclo/VCR maintenance) Arm 2: Standard treatment + RT with Carbo Arm 3: Standard treatment + isotretinoin during maintenance Arm 4: Standard treatment + RT with Carbo + isotretinoin during maintenance	3–21 yrs High-risk MB	261	Survival advantage for Grp 3 MB receiving RT with carboplatin. 5 yr EFS/OS: 73.2%/82.3% vs. 53.7%/63.7% Isotretinoin therapy futile
High-Grade Gliomas					
2004– 2005	ACNS0126 [54]	RT (HGG 54 Gy, DIPG 59.4 Gy) + concomitant low-dose TMZ, followed by 10 cycles of higher dose TMZ continuation therapy	Age 3–≤22 yrs	HGG = 107 DIPG = 63	1 yr EFS/OS 14%/40% No improvement vs. historical controls
2005– 2007	ACNS0423 [55]	RT (GTR 54 Gy, STR 59.4 Gy, spinal cord lesions 50.4–54 Gy) + concomitant low-dose TMZ, followed by up to 6 cycles of higher dose TMZ + CCNU continuation	Age 3–≤22 yrs	108	3 yr EFS/OS 22%/19% Improved vs. ACNS0126
2007– 2008	ACNS0222 [56]	RT (54 Gy) with motexafin-gadolinium as a potent radiosensitizer	Age ≤ 21 yrs Unifocal DIPG	60	1 yr EFS/OS 18%/53% No Improvement
2011– 2015	HERBY [57]	Randomization Arm 1: RT (54 Gy) + low-dose TMZ, continuation high-dose TMZ 12 months Arm 2: RT (54 Gy) + low-dose TMZ + Bev, continuation high-dose TMZ + Bev 12 mnths	Age ≥ 3–≤18 yrs Non–brainstem	116	1 yr median EFS 11.8 vs. 8.2 mnths No improvement
2014– 2020	BIOMEDE 1 [58]	Randomization Arm 1: RT + Everolimus Arm 2: RT + Dasatinib Arm 3: RT + Erlotinib	Age 6 mths–25 yrs DIPG	193	Median OS Arms 1, 2, 3 10.9, 9.5 and 9 mnths No improvement
Ependymoma					
2003– 2007	ACNS0121 [59]	Stratum 1: Completely resected differentiated, ST ependymoma undergo observation Stratum 2: Incompletely resected ependymoma undergo chemotherapy, second surgery and RT Stratum 3: Near-total or macroscopic GTR undergo conformal RT Stratum 4: Microscopic GTR undergo conformal RT, excluding differentiated, ST lesions	Age 1–21 yrs	356	5 yr EFS/OS Strata 1: 61%/100% Strata 2: 37.2%/70.2% Strata 3: 67%/83.3% Strata 4: 70%/88.3%
2010– 2017	ACNS0831 [60]	PF tumours gross/near total resection: randomization Arm 1: RT alone Arm 2: RT + 4 cycles VCR/Cis/Cyclo/VP16	Age 1–21 yrs	451	3 yr EFS 71% vs. 80% ? chemotherapy superior

RT: radiotherapy; CSI: craniospinal irradiation; PF: posterior fossa; Carbo: carboplatin; VP16: etoposide; Cyclo: cyclophosphamide; MB; medulloblastoma; EFS: event-free survival; VCR; vincristine; CCNU: lomustine; Cis: cisplatin; HFRT: hyper-fractionated radiotherapy; STRT: standard radiotherapy; TB: tumor bed; OS: overall survival; SR: standard risk; HR: high risk; MTX: methotrexate; LR: low risk; IR: intermediate risk; Topo: topotecan; CR: complete response; CCR: continuous complete response; PRD: persistent residual disease; Ifos: ifosfamide; GTR: gross total resection; DIPG: diffuse intrinsic pontine glioma; yrs: years; mnths: months.

**Table 2:** Future clinical challenges for pediatric malignant brain tumors.

Tumor Group	Future Clinical Challenge
ALL	Modernize trial risk stratification according to biology Improve trial design to allow timely conclusions across smaller patient populations Enable multinational trial collaboration, including less affluent countries Discovery of novel agents with rapid pre-clinical to clinical translation Improved understanding of, and therapies for, recurrence (need for repeat tissue analysis via surgery, etc.) Awareness of neuro-disability, quality of survival and protracted follow-up in trial designs
Medulloblastoma	
WNT	Non-metastatic; de-escalation of therapy
SHH	Metastatic/MYCN amplified/TP53 mutant; therapy intensification or novel agent(s)
Group 3	MYC amplified and/or metastatic; therapy intensification or novel agent(s)
Group 4	Non-metastatic and chromosome 11 loss; de-escalation of therapy Metastatic; intensification or novel agent(s)
High-grade gliomas	Mandating tissue analysis of brainstem lesions for trial entry International collaborative efforts to test novel agents for specific molecular subgroups Consideration of alternative drug delivery methods, e.g., convection enhanced delivery
Ependymoma	
PF-A	Chromosome 1q gain +/− 6q loss; novel agents(s) or techniques including increased radiosensitization
PF-B	Chromosome 13q balanced; de-escalation of therapy
ST-ZFTA	Stratification of therapy dependent on extent of surgical resection
ST-YAP1	De-escalation of therapy
